# A Scoring System for Identifying Patients Likely to Be Diagnosed with Low-Grade Coeliac Enteropathy

**DOI:** 10.3390/nu11051050

**Published:** 2019-05-10

**Authors:** Fernando Fernández-Bañares, Anna Carrasco, Mercè Rosinach, Beatriz Arau, Roger García-Puig, Clarisa González, Eva Tristán, Yamile Zabana, Maria Esteve

**Affiliations:** 1 Department of Gastroenterology, Hospital Universitari MutuaTerrassa, 08221 Terrassa, Barcelona, Spain; anna.carrasco.garcia@gmail.com (A.C.); mariamercerosinach@gmail.com (M.R.); beatrizarau@mutuaterrassa.es (B.A.); etristan@mutuaterrassa.cat (E.T.); yzabana@gmail.com (Y.Z.); mariaesteve@mutuaterrassa.cat (M.E.); 2 Centro de Investigaciones Biomédicas en Red de enfermedades hepáticas y digestivas (CIBERehd), 28029 Madrid, Spain; 3 Department of Paediatrics, Hospital Universitari MutuaTerrassa, 08221 Terrassa, Barcelona, Spain; rgarcia@mutuaterrassa.cat; 4 Department of Pathology, Hospital Universitari MutuaTerrassa, 08221 Terrassa, Barcelona, Spain; cgonzalez@mutuaterrassa.es

**Keywords:** prediction, response to treatment, coeliac disease, IgA anti-transglutaminase 2 deposits

## Abstract

Background & Aims: Determining whether patients with lymphocytic enteritis (LE) have coeliac disease is a challenge. We analysed the variables associated with a low-grade coeliac enteropathy diagnosis in patients with suspected coeliac disease but without villous atrophy, and developed a scoring system to identify them. Methods: We collected data from 2010 through to 2016 on patients with lymphocytic enteritis and persistent symptoms compatible with the clinical spectrum of coeliac disease. One hundred and four patients starting on a gluten-free diet (GFD) were included. Duodenal biopsies were collected before the GFD and analysed for numbers of CD3^+^ T-cell receptor gamma delta+ (TCRγδ^+^), and CD3^−^ intraepithelial lymphocytes. We performed a logistic regression analysis to identify factors associated with a low-grade coeliac enteropathy diagnosis. Results: Sixty-two patients achieved clinical remission after the GFD. Fifty of these 62 patients were diagnosed with low-grade coeliac enteropathy. Multivariate analysis identified the presence of >25% intraepithelial lymphocytosis, HLA-DQ2.5, positive serology, and increased numbers of TCRγδ^+^ cells with a low-grade coeliac enteropathy diagnosis. We developed a scoring system that identified patients with an area under the ROC curve (AUC) of 0.91. Scores of >10 had 86% sensitivity and 85% specificity. Conclusion: We developed a scoring system that identifies patients likely to be diagnosed with low-grade coeliac enteropathy with an AUC value of 0.91.

## 1. Introduction

The spectrum of the so-called gluten-related disorders has expanded beyond well-recognised conditions such as coeliac disease (CD) and dermatitis herpetiformis, and now encompasses other disorders whose aetiologies have been connected to gluten [1]. Chief among these is non-coeliac gluten sensitivity (NCGS), which has been diagnosed in individuals who do not have CD or wheat allergy but whose clinical symptoms are dependent on gluten [1]. However, certain patients with NCGS may present with some of the features associated with CD though insufficient to justify that diagnosis. Milder lesions (Marsh 1) are nonspecific since only 10% of the subjects presenting with this pattern prove to have CD [2], while NCGS patients may have associated intraepithelial lymphocytosis [1]. Positive serology increases the likelihood of CD; however, under these circumstances, the sensitivity of serology is very low [2]. Some symptomatic patients with increased intraepithelial lymphocytes (IEL) have intestinal tissue transglutaminase deposits (IgA anti-tTG2 deposits), although they do not have circulating antibodies against tTG2 [3]. In addition, some patients exhibiting an increase in IEL express CD3+ T-cell receptor gamma delta+ (TCRγδ^+^), which are considered to be suggestive of CD [4]. Furthermore, these patients typically carry the same HLA risk alleles as patients with CD [2]. Therefore, a subset of patients with gluten sensitivity might actually belong to the clinical spectrum of CD. A number of authors have considered that these patients present a mild form of CD, and have used the terms “coeliac-light”, “coeliac-lite”, “celiac trait”, “mild enteropathy CD”, or “low-grade gluten-sensitive enteropathy” to refer to them [2,5,6,7,8,9]. Previous studies have shown that these patients have the same clinical manifestations as patients with CD [8,9,10,11,12]. In addition, we have shown that a blinded gluten challenge in these patients was associated with a significantly higher clinical relapse rate and a deterioration in the quality of life as compared with placebo, reinforcing the role of gluten in the pathogenesis of this mild enteropathy [13].

In this complex scenario, the cut-off level used to define the upper limit of normal IEL/100 epithelial cells is controversial and far from standardised [14]. In fact, the reported upper limit of the normality varies from 18 to 40 IEL/100 epithelial cells [15,16,17,18]. Previous studies have suggested that routine duodenal studies should include TCRγδ^+^ cell counts in order to detect CD in these cases [4,19,20,21,22]. In addition, subepithelial IgA tTG (tissue transglutaminase) deposits have been considered to be a promising tool for CD diagnosis in doubtful cases [23,24].

The present study aimed to identify the clinical, histological, genetic, and immunological variables associated with a low-grade coeliac enteropathy diagnosis, and to derive a scoring system that can be used to identify these patients.

## 2. Patients and Methods

### 2.1. Patients

From April 2010 to September 2016, all patients from whom duodenal biopsies were taken to rule out CD were prospectively recorded. The indications for duodenal biopsy sampling were long-standing gastrointestinal or extraintestinal symptoms suggestive of CD and positive coeliac serology. In addition, most patients were referred for duodenal biopsies on the additional basis of positive HLA-DQ2.5/8, mainly those with negative coeliac serology.

We included patients based on the following inclusion criteria: (1) undergoing duodenal biopsies performed while on a gluten-containing diet; (2) the presence of duodenal intraepithelial lymphocytosis and the absence of villous atrophy; and (3) starting on a gluten-free diet (GFD), with either a well-defined response (see below) or no response to the diet. Patients were excluded if: (1) follow-up biopsies were not accepted; (2) there was a clinical but not a histological response to GFD in patients with negative coeliac serology; (3) the presence of *Helicobacter pylori* (*Hp*) infection (these patients could be included if, following eradication, there was persistence of symptoms compatible with the CD spectrum and a new duodenal biopsy was performed with persistent duodenal intraepithelial lymphocytosis); (4) the intake of nonsteroidal anti-inflammatory drugs and angiotensin II receptor blockers at index endoscopy; (5) the presence of intestinal parasitic infection; and (6) a final diagnosis of inflammatory bowel disease, microscopic colitis, or another specific enteropathy. In relation to exclusion criterion number two, seronegative patients with clinical but not histological remission after a GFD were not included since they were considered unclassifiable for the present study. Therefore, since the study was designed to identify the factors associated with a low-grade coeliac enteropathy diagnosis, only patients with a clear clinical plus histological (or serological) response to GFD likely to be diagnosed with low-grade coeliac enteropathy (true positives) or with a clear non-response to a six-week GFD (true negatives) were included.

A GFD was administered to all included patients based on a clinical picture of the CD spectrum, positive serology, positive coeliac genetics, and a CD IEL cytometric pattern [11,13,22]. A GFD was administered by personnel at the specialised coeliac outpatients’ clinic. Assessment by a dietician of diet compliance was requested when there was a suspicion of non-adherence in the direct clinical interview with the patient during the three-month follow-up visit. Afterwards, visits were scheduled every six months during all of the follow-up periods.

The study protocol was approved by the Ethics Committee of the “Hospital Universitari MútuaTerrassa”, and all participants provided informed consent.

### 2.2. Studies on Duodenal Biopsies

Two endoscopic biopsies from the bulb and four from the second portion of the duodenum were obtained and placed in separate vials using 2.8 mm biopsy forceps (Radial Jaw^TM^ 4, Boston Scientific, USA) for standard histological studies of the index endoscopy before the GFD. One additional biopsy for IEL flow cytometry and two for intestinal anti-transglutaminase 2 immunoglobulin A deposits (IgA tTG2 deposits) were obtained from the second duodenal portion. In addition, two antral biopsies were taken to assess the presence of *Hp* infection. IEL counts were performed as previously described [25]. Lymphocytic enteritis (LE) was defined as 19 or more IELs per 100 epithelial nuclei and normal villous architecture, based on the lower cut-off of normality described in the literature [17]. Patients were further classified, based on the IEL count, into those with 19 to 25 IELs and those with >25 IELs per 100 epithelial nuclei.

The sample for IEL flow cytometry was immediately processed, as previously described by our group [13,22], to assess the numbers of CD3^+^ T-cell receptor gamma delta+ (TCRγδ^+^) and CD3^−^ intraepithelial lymphocytes. IgA tTG2 deposits were also assessed as previously described [13,22]. A brief methodological description of the two procedures is described in Supplementary File 1.

### 2.3. Coeliac Serology

Serum IgA anti-tTG2 (or IgG anti-tTG2 in IgA-deficient patients) was analysed using a quantitative automated ELISA (Elia CelikeyTM, Phadia AB, Freiburg, Germany) with recombinant human tTG2 as antigen (positive values >2 U/mL) [25]. Anti-tTG2 titres between 2 and 8 U/mL were considered as positive only if confirmed by positive endomysial antibody (EmA). EmA was performed by indirect immunofluorescence assay in serum samples at 1:5 dilution (commercial sections of monkey distal oesophagus; BioMedical Diagnostics, Marne-la-Vallée, France) in all patients with positive tTG2. Total serum IgA was measured using rate nephelometry (BN II, Siemens Healthcare Diagnostics SL, Marburg, Germany).

### 2.4. Coeliac Genetics

Methods of assessment of coeliac genetics are described in Supplementary methods.

### 2.5. Low-Grade Coeliac Enteropathy Diagnosis

As mentioned above, the response to GFD was defined as both clinical remission and the serological or histological response to the diet. LE with positive serum anti-tTG2 antibodies was considered to be indicative of low-grade coeliac enteropathy if there was a clinical and serological response to the GFD. Additionally, seronegative LE was considered to be indicative of low-grade coeliac enteropathy if there were typical symptoms of CD at presentation, when there was both clinical remission and histological response to a GFD and finally, in cases of clinical relapse after gluten reintroduction (at least, 10 g per day).

Clinical improvement after a strict GFD was evaluated at 1.5, 3, 6, and 12 months. Clinical remission was considered to have occurred when there was a complete resolution of symptoms and a normalisation of abnormal analytical parameters (haemoglobin, iron, transaminases), which was maintained at the 12-month follow-up visit (in the case of iron-deficiency without requiring iron supplements). In patients with a sustained clinical response to a GFD, a follow-up biopsy was performed at least 12 months after starting the diet to assess the histological response. Histological remission was considered to have occurred when there was a normal IEL count (<19% IELs) or a reduction of at least 50% from baseline in the follow-up biopsy [25]. A reduction in the IEL count ≥30% [26], and <50% from the basal biopsy was considered to be a histological response.

### 2.6. Statistical Analysis

Results are expressed as mean ±SEM and as proportions. Chi-square statistics were used to compare qualitative variables, and the Student *t*-test was used to compare quantitative variables. Those variables with a significant association in the univariate analyses (*p* < 0.10) were introduced into a multivariate model for logistic regression analysis. This analysis was performed to assess the association between possible predictors and the diagnosis of low-grade coeliac enteropathy (yes/no). A rule of thumb of 10 events per variable was used to obtain the minimum sample size, assuming that the logistic regression model may account for four dummy predictor variables. In this line, assuming a 40% response rate to a GFD, a minimum sample size cohort of 100 individuals was required. A stepwise method of introduction was used. The odds ratio (OR) and its 95% confidence interval (CI) were calculated. For each risk factor, we assigned a weight in the risk score using the respective OR yielded by the logistic regression, where the maximum log-OR received a score of 10 points. Receiving operator curves (ROC) and the Youden index were used to define the best cut-off point for the new score. Accuracy was measured using the area under the ROC curve (AUC). Bootstrap estimation of the AUC 95% CI (3000 stratified replicates) was performed as an internal validation assessment. Sensitivity, specificity, positive and negative likelihood ratios (LR+ and LR−), and their 95% confidence intervals (CI), were calculated. We took LR+ >10 and LR− <0.1 as convincing diagnostic evidence, while values >5 and <0.2, respectively were seen as moderate diagnostic evidence.

Statistical calculations were performed using the SPSS for Windows statistical package (SPSS Inc., Chicago, IL, USA), except for the ROC analysis, for which the Library “pROC” was used [27]. Statistical significance was predetermined as *p* < 0.05.

## 3. Results

**Figure 1:**
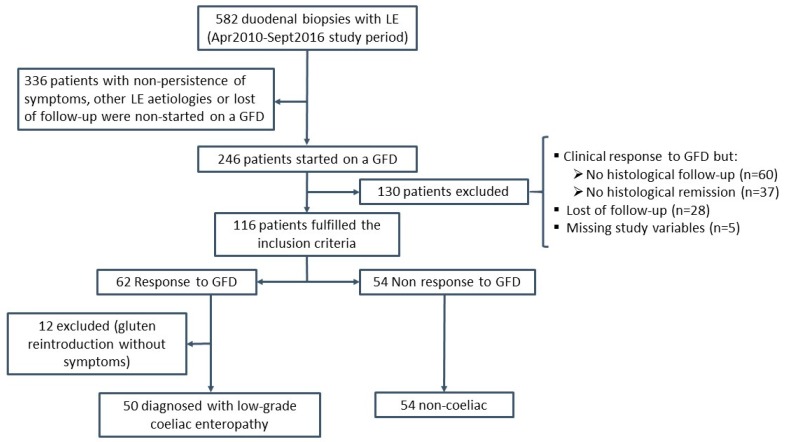
Flow diagram of the participants throughout the study. LE: lymphocytic enteritis (IEL count >19%); GFD: gluten-free diet.

The flow of patients during the study is presented in Figure 1. One-hundred and sixteen patients fulfilled the inclusion criteria (72% women; mean age 39 ± 1.5 years). At inclusion, 62 patients showed a response to the GFD and 54 showed no response. The clinical picture of patients before starting the GFD is described in Supplementary Table S1, according to whether they had presented a response to GFD or not. As mentioned above, patients with a response to the GFD were symptom-free and had normalised abnormal analytical parameters after a GFD, which had been maintained for at least 12 months of follow-up. In addition, 89% of these patients had presented histological remission and 11% a histological response (Supplementary Figure S1). Fifty out of the 62 patients (81%) with a response to the GFD were diagnosed with low-grade coeliac enteropathy: 20/50 (40%) achieved clinical, serological, and histological remission, 26/50 (52%) achieved clinical and histological remission, and 4/50 (8%) achieved clinical remission and a histological response with clinical relapse after gluten reintroduction. Nine out of the other 12 patients with a response to GFD achieved clinical and histological remission, and three achieved clinical remission and histological response. However, gluten reintroduction did not induce symptom relapse in any of these patients, who were excluded from the analysis since the final diagnosis was unclear.

**Figure 2:**
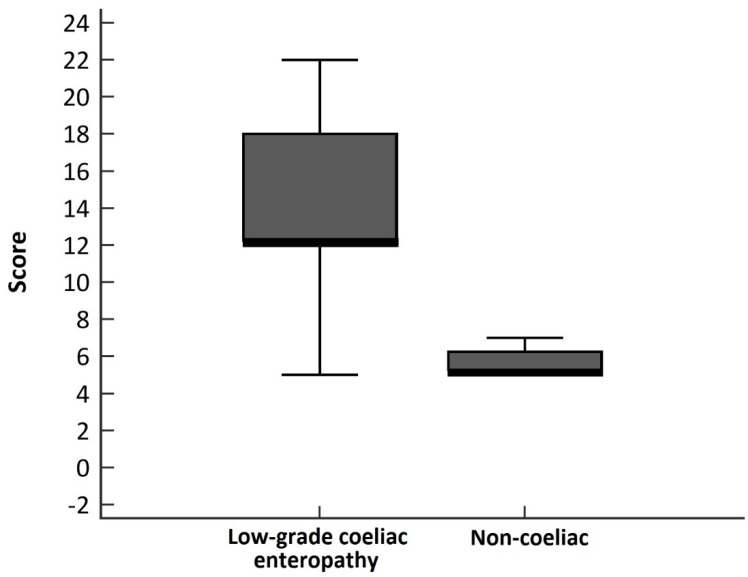
Boxplot chart showing the distribution of the derived score by final diagnosis.

The demographic, clinical, histological, and biological characteristics of the included patients before starting the GFD according to the final diagnosis are presented in Table 1. A multivariate-adjusted logistic regression analysis demonstrated that the presence of serum anti-tTG2 > 2 U/mL, an increase in CD3^+^ TCRγδ^+^ cells, and LE (> 25% IEL) were independently associated with a low-grade coeliac enteropathy diagnosis (Table 2). Since we had a special interest in seronegative patients, the analysis was repeated excluding serology as a predictor variable. The results were similar but included the presence of HLA-DQ2.5 as a predictor variable (Table 2). A scoring system was derived taking into account these variables, which ranged from −2 to 25 points (Table 3). The median score was eight points (IQR, 6.2–15). Figure 2 describes the distribution of the derived score in low-grade coeliac enteropathy and non-coeliac patients.

**Figure 3:**
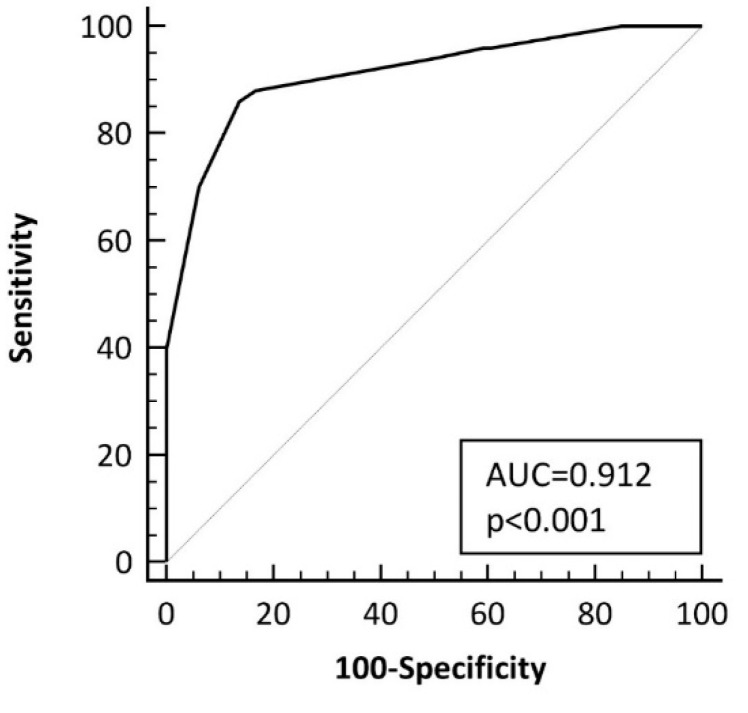
Curve for the accuracy of the scoring system at identifying patients with low-grade coeliac enteropathy. AUC: area under the curve, ROC: receiver operating characteristic.

The optimal cut-off point of the score was of >10 points, which yielded an AUC of 0.91 ± 0.03 (bootstrapped 95% CI, 0.84 to 0.96) (Figure 3), with a sensitivity of 86% (95% CI, 73–94%) and a specificity of 85.2% (95% CI, 73–93%) for low-grade coeliac enteropathy diagnosis. The accuracy of the different criterion values and the coordinates of the ROC curve are described in Supplementary Table S2. The derived score was associated with an OR of 35 (95% CI, 12–105) for identifying patients with low-grade coeliac enteropathy (Score ≤ 10, 7 out of 53, 13.2%; Score > 10, 43 out of 51 patients, 84.3%; *p* < 0.0001).

When selecting patients with low-grade coeliac enteropathy in clinical and serological remission (*n* = 20) and comparing them to the non-coeliac patients (*n* = 54), the derived score system yielded an AUC of 1 for the identification of patients with low-grade coeliac enteropathy. When selecting low-grade coeliac enteropathy seronegative patients with clinical and histological remission/response (*n* = 30), the AUC was 0.85 ± 0.04 (Supplementary Figure S2), and the cut-off point of the score of >10 points provided a sensitivity of 77% (95% CI, 58–90%) and a specificity of 85% (95% CI, 73–93) (see the accuracy of the different criterion values and the coordinates of the ROC curve in Supplementary Table S2). The results were similar after excluding low-grade coeliac enteropathy patients with only a histological response (*n* = 26) (AUC, 0.8 4 ± 0.05).

### 3.1. Intestinal IgA Transglutaminase 2 Subepithelial Deposits

The presence of IgA tTG2 deposits was evaluated in 104 patients, though 20 (19%) of them were excluded because of a non-conclusive result due to technical failure. IgA tTG2 deposits were positive in 50 out of the 84 (59.5%) included patients, being positive more often in patients with low-grade coeliac enteropathy (30 out of 40; 75%) than in non-coeliac patients (20 out of 44; 45%) (*p* = 0.006). There was an interaction between IgA tTG2 deposits and serum-anti-tTG2, since positive serum anti-tTG2 was more frequent in patients with positive deposits (IgA tTG2 deposits+, 36% vs. IgA tTG2 deposits-, 9%; *p* = 0.005).

Finally, a binary regression logistic analysis including IgA tTG2 deposits and the derived score system revealed that only the score system was associated with a low-grade coeliac enteropathy diagnosis (OR, 48; 95% CI, 13–182; *p* < 0.001) (IgA tTG2 deposits: OR, 2.1; 95% CI, 0.5–8.2; *p* = 0.3).

### 3.2. Accuracy for the Diagnosis of Seronegative Low-Grade Coeliac Enteropathy

We evaluated the diagnostic accuracy of the various studied parameters for low-grade coeliac enteropathy diagnosis in seronegative patients, for whom the diagnosis is most challenging. As shown in Table 4, the derived score system was the most accurate parameter in these cases.

## 4. Discussion

We developed a scoring system that identifies patients likely to be diagnosed with low-grade coeliac enteropathy with an AUC value of 0.91. The derived score may be especially useful for diagnosing those patients with positive serum anti-tTG2 at low titres and in the seronegative patients. In this setting, the terminology is confusing. The term CD is used for patients with villous atrophy. Furthermore, the term potential CD is used for individuals with normal small intestinal mucosa who are at an increased risk of developing CD as indicated by positive CD serology [28]. In this context, some authors consider that having an increased number of IELs in the villi is not a pathological condition [28]. These “potential patients” are frequently not treated as coeliacs with a GFD, and this is troubling since both our own and other previous studies have shown that these patients may present with intestinal and extraintestinal symptoms compatible with the CD clinical spectrum which improves after a GFD [8,9,10,11,12]. In this sense, it has been argued that this entity represents the milder domain of the CD spectrum, though the use of several different terms to identify it, such as “coeliac-light”, “coeliac-lite”, “celiac trait”, “mild enteropathy celiac disease”, or “low-grade gluten-sensitive enteropathy” [2,5,6,7,8,9], has contributed to a degree of misunderstanding. In this paper, we decided to use the term low-grade coeliac enteropathy to refer to these patients, which is a combination of proposed terms and avoids the use of “gluten sensitivity” that can create confusion with NCGS.

As mentioned, serology is often negative in mild forms of CD [2,29,30]. There is, therefore, an urgent need for new tools for the diagnosis of either CD or low-grade coeliac enteropathy when serology is negative. In this sense, it has been described that the number of TCRγδ^+^ IELs, a typical feature of CD histopathology, is only elevated in CD subjects, while in NCGS patients, the number of TCRγδ^+^ IELs is similar to that in controls [31]. Assessment of the density of TCRγδ^+^ IEL is, in general, carried out using immunohistochemistry techniques on frozen biopsy samples [32,33]. This is a laborious user-dependent technique in well-orientated high-quality samples, but sampling is often compromised, and conclusions may be difficult to draw. The assessment of TCRγδ^+^ IEL with flow cytometry offers substantial advantages [20,21,22]. It allows for the analysis of a greater number of cells than immunohistochemistry and results are obtained in a fast, sensitive, reproducible, and objective way just a few hours after taking the biopsy sample. The concomitant measurement of CD3^−^ IEL adds specificity to the assay as shown in the present and previous studies [21,22]. In view of these results, it seems important to take into account that taking an additional biopsy for flow cytometry is an easy procedure that may yield substantial information. Most laboratories in tertiary and even secondary hospitals dispose of a flow cytometer for diagnostic purposes and analysing the lymphocyte subpopulations in the duodenal mucosa is an affordable technique.

Subepithelial IgA tTG2 deposits have been considered to be a promising tool for diagnosing CD in doubtful cases [23,24]. However, this technique presents the same pitfalls as the immunohistochemistry of TCRγδ^+^ cells discussed above. Non-conclusive results occurred in 19% of our samples, limiting its usefulness in clinical practice. In this sense, we decided not to include this variable in the initial logistic regression analysis to maintain an appropriate sample size. Further analysis in a subsample revealed that only the derived score system was associated with a low-grade coeliac enteropathy diagnosis. Moreover, although IgA tTG2 deposits were more frequently positive in seronegative low-grade coeliac enteropathy patients than in non-coeliac ones, their specificity was low, precluding their clinical use in this setting. Our results, therefore, suggest that intestinal IgA tTG2 deposits do not provide relevant information for the diagnosis of low-grade coeliac enteropathy. In this sense, recent studies also suggest that IgA tTG2 deposits do not show absolute specificity for CD [34].

In relation to the need to confirm the cut-off level for defining the upper limit of normal IEL/100 epithelial cells, the results of the current study suggest that a cut-off of 25% IEL significantly increases the probability of low-grade coeliac enteropathy. However, there were some patients with lower cut-offs who were also diagnosed with low-grade coeliac enteropathy and this limit cannot, therefore, be interpreted to be an absolute rule.

Our study has some limitations related to the patient sample evaluated since low-grade coeliac enteropathy was suspected in all included patients. In fact, 80% were positive for HLA-DQ2/8, and only 4% were negative for the two alleles DQ2.5 and DQ8. Thus, the performance of the scoring system in a different clinical scenario should be evaluated. Although the scoring system was internally validated using bootstrapping techniques, further studies for external validation in different clinical practice settings will be needed to generalise its diagnostic use.

In conclusion, we developed a scoring system that identifies patients with LE and with long-term clinical symptoms compatible with the coeliac spectrum, positive HLA-DQ2.5, and coeliac serology, and an increase in intraepithelial TCRγδ+ cells which are likely to be diagnosed with low-grade coeliac enteropathy with an AUC value 0.91. Intestinal IgA tTG2 subepithelial deposits do not provide relevant information for the diagnosis of these patients.

## Figures and Tables

**Table 1:** The clinical and biological characteristics of the included patients according to the final diagnosis.

Variable	Low-Grade Coeliac Enteropathy (*n* = 50)	Non-Coeliac (*n* = 54)	*p*
Sex (% women)	30 (60%)	43 (79.6%)	0.029
Age (years)	37.3 ± 2.8	41.3 ± 1.9	0.24
CD 1st degree relatives (%)	13 (26%)	9 (16.6%)	0.24
Clinical presentation			
Gastrointestinal	42 (84%)	48 (89%)	0.46
Extraintestinal	8 (16%)	6 (11%)	
IEL count (per 100 epithelial cells)	39.0 ± 1.8	37.2 ± 1.8	0.46
19–25 (per 100 epithelial cells) (%)	5 (10%)	13 (24%)	0.06
>25 (per 100 epithelial cells) (%)	45 (90%)	41 (76%)	
HLA-DQ2.5/8+ (%)	45 (90%)	38 (70.4%)	0.013
DQ2.5+ (%)	38 (76%)	23 (42.6%)	0.001
DQ8+ (%)	11 (25%)	19 (37.3%)	0.20
DQ8+ plus DQ2.2+ (%)	2 (4%)	0 (0%)	0.23
2 alleles of DQ2.5- and DQ8- (%)	0 (0%)	4 (7.4%)	0.11
Serum anti-tTG2 >2 (%)	20 (40%)	1 (1.9%)	<0.0005
>30 U/mL	3	0	
>8 to 30 U/mL	10	0	
>2 to 8 U/mL *	7	1	
IEL cytometry pattern			
↑TCRγδ^+^ (%)	45 (90%)	15 (27.8%)	<0.0005
↑TCRγδ^+^ plus↓CD3^−^ (%)	27 (54%)	7 (13%)	<0.0005

* EmA+ in five out of seven patients with low-grade coeliac enteropathy, and in zero out of one non-coeliac. CD: Coeliac disease; IEL: intraepithelial lymphocytes; Anti-tTG2: IgA anti-transglutaminase antibodies; TCRγδ^+^: CD3+ T-cell receptor gamma delta+.

**Table 2:** Multivariate-adjusted logistic regression model (*n* = 104): Predictor variables of low-grade coeliac enteropathy diagnosis.

Predictors	OR (95% CI)	*p* Value
Serum anti-tTG2 >2 U/mL	53 (2.5–1102)	0.010
↑TCRγδ^+^ IEL	26.4 (8–92)	<0.0005
IEL count >25%	15.5 (2.8–86)	0.002
**After excluding serology as a predictor variable:**		
↑TCRγδ+ IEL	36.1 (10–126)	<0.0005
IEL count >25%	7.85 (2–32)	0.004
HLA-DQ2.5+	3.2 (1.03–10)	0.043

IEL: intraepithelial lymphocytes; Anti-tTG2: IgA anti-transglutaminase antibodies; TCRγδ^+^: CD3+ T-cell receptor gamma delta+.

**Table 3:** Assigned points for the predictors and derived scoring system (−2 to 25 points).

Predictors	Points
Serum anti-tTG2	
>20 U/mL	10
>8–20 U/mL or >2–8 U/mL *plus*EmA+	6
>2 to 8 U/mL *plus*EmA-	2
≤2 U/mL	0
IEL cytometry pattern	
↑TCRγδ^+^ cells	7
Histology (IEL count)	
>25%	5
19–25%	0
<19%	−1
Coeliac genetics:	
DQ 2.5+	3
DQ8+/DQ2.2+/Allele DQB1 of haplotype DQ2.5+	0
2 alleles DQ2.5- and DQ8-	−1

Serum anti-tTG2: IgA anti-transglutaminase antibodies; EmA: IgA anti-endomysium antibodies; IEL: intraepithelial lymphocytes; TCRγδ^+^: CD3+ T-cell receptor gamma delta+.

**Table 4:** The accuracy of a score >10 as compared to the increase of TCRγδ^+^ IEL, positive CD lymphogram (increase in TCRγδ^+^ plus a decrease in CD3^−^ IELs), and positive IgA tTG2 deposits for low-grade coeliac enteropathy diagnosis in seronegative patients (*n* = 84 seronegative patients; 30 low-grade coeliac enteropathy vs. 54 non-coeliac).

	Sensitivity (%) (95% CI)	Specificity (%) (95% CI)	LR+ (95% CI)	LR− (95% CI)
**Score system >10**	77 (CI, 57–89)	85 (CI, 72–93)	5.2 (CI, 2.6–10)	0.27 (CI, 0.14–0.53)
**↑TCRγδ+**	83 (CI, 64.5–94)	72 (CI, 58–83)	3.0 (CI, 1.9-–4.7)	0.23 (CI, 0.1–0.52)
**CD lymphogram**	43 (CI, 26–62)	87 (CI, 74–94)	3.3 (CI, 1.5–7.5)	0.65 (CI, 0.5–0.89)
IgA tTG2 deposits *	65 (CI, 41–84)	54.5 (CI, 39–69)	1.4 (CI, 0.9–2.2)	0.64 (CI, 0.34–1.2)

* *n* = 64 (20 low-grade coeliac enteropathy vs. 44 non-coeliac), since patients with non-conclusive results were excluded from the analysis.

## Supplementary Materials

The following are available online at https://www.mdpi.com/2072-6643/11/5/1050/s1. Supplementary methods: Flow cytometry, intestinal deposits of anti-TG2 IgA antibodies, and HLA genotyping. Table S1: Baseline clinical picture of the included patients as a function of their GFD response. Table S2: Accuracy of the different criterion values and coordinates of the ROC curve in both the overall series of patients, and after excluding patients with positive serum anti-tTG2. Figure S1: Evolution of the intraepithelial lymphocyte count (IEL count per 100 epithelial nuclei) in patients with clinical and histological remission/response after a GFD. Figure S2: ROC curve for the accuracy of the scoring system at identifying patients with low-grade coeliac enteropathy after excluding patients with positive serum anti-tTG2. AUC: area under the curve, ROC: receiver operating characteristic.

## Writing Assistance

David Bridgewater, Anglocatalan Services, Barcelona.
