# Supplementary material for: A Scoring System for Identifying Patients Likely to Be Diagnosed with Low-Grade Coeliac Enteropathy
Source: Nutrients. 2019 May 10;11(5):1050. doi: 10.3390/nu11051050 (PMC6566304; doi:10.3390/nu11051050)
Supplement: Supplementary file 1 [file nutrients-11-01050-s001.pdf]

Supplementary methods

Flow cytometry

One single duodenal biopsy was obtained using a 2.8 mm biopsy forceps (Radial Jaw 4, Boston Scientific, USA), and immediately processed as previously described with minor modifications[1]. Preparations of IEL suspensions were performed by incubation with 1 mM EDTA, 1 mM DTT in HBSS for 90 minutes with continuous rotation at 12 rpm in a vertical shaker at room temperature. This procedure achieved the total removal of villous epithelium and the partial removal of crypt epithelium. The proper separation of epithelial compartment was confirmed by an immunohistochemical analysis of the remaining tissue during the protocol validation. The obtained suspension, a mixture of IEL and epithelial cells, was washed once in fresh HBSS at 1500 rpm for 10 minutes, and IEL were immediately stained with previously titrated amounts of directly labelled antibodies for 15 minutes at room temperature. The antibodies used to define the different IEL subsets were anti-CD45-APC (clone 2D1), anti CD3-PerCP (clone SK7), anti CD103-FITC (clone Ber-ACT8), and anti –TCR γδ-PE (clone 11F2) (all from BD Biosciences, Franklin Lakes, NJ, USA). The intraepithelial origin of the IEL suspension was verified with CD103+ staining, and it was always ≥85%. Cells were immediately analysed on a standard 4-color FACSCalibur instrument (BD Biosciences, Franklin Lakes, NJ, USA). Cell counts of the recovered cell number for biopsy were performed with a hemocytometer and trypan blue exclusion.

Results were obtained 3 to 4 hours after biopsy sampling, and expressed as percentages of bright CD45 staining and a low sideward scatter gate. The normal cut-off values for the IEL cytometric pattern in our laboratory are CD3+TCRγδ+ IEL, 8.5% (<mean+2SD) and CD3- IEL >10% (10th percentile). The cut-offs were calculated in a sample of 65 non-coeliac subjects. The intra-assay coefficient of variation was 5.5% (two replicates of each sample processed one immediately after the other), and the inter-sample coefficient of variation was 7.7% (two different samples from each patient obtained in the same procedure).

Intestinal deposits of anti-TG2 IgA antibodies

One biopsy specimen from 2nd-3rd duodenal portion was embedded in an optimal cutting temperature compound (OCT-Tissue-Tek, Sakura, Finetek, Netherland) and immediately frozen in liquid nitrogen. Frozen biopsy samples were stored at -80ºC until used. Six sections (5 µm) from each patient were examined by double immunofluorescence for intestinal deposits of anti-tTG2 IgA antibodies. The staining protocol was performed in a humid chamber at room temperature. The sections were first incubated with a mouse monoclonal antibody against tTG2 (clone CUB7402) (dilution 1:200, 15 minutes; Abcam, Cambridge, UK). The sections were washed in PBS and incubated with a secondary antibody anti-mouse-Alexa 594 conjugated (red) (1:200, 15 minutes) from Invitrogen (Paisley, UK). Slides were then washed again and stained with FITC-labelled Rabbit polyclonal antibody to human IgA (green) (1:40, 30 minutes). Samples from IgA deficient patients were incubated with polycolonal rabbit anti-human IgG under the same conditions. Both antibodies were from Dako (Denmark). Finally, the sections were washed twice in PBS and mounted with a fluorescent mounting medium (Dako, Denmark). The colocalization images of IgA mucosal deposits and TG2, which appeared in yellow/orange, were analysed with a confocal microscope (SP2, Leica, Germany). The evaluation of anti-TG2 IgA deposits was performed considering the pattern and the intensity of the staining as described[1]. Positive deposits were considered to be of either low (+) or high (++) intensity. In our previous study, 2 of the 10 healthy controls (20%; CI, 5.6% to 50%) with negative HLA-DQ2/8 haplotypes showed low intensity positive deposits. In other previous studies, non-coeliac control subjects also presented positive mucosal deposits in 12 to 20% of cases (see reference 1).

HLA genotyping

Genomic DNA from whole blood was purified using the commercial Qiamp DNA Blood Mini kit (Qiagen, Düsseldorf, Germany). A commercial reverse hybridization kit for the detection of CD heterodimers HLA-DQ2.5 (A1*0501/*0505, B1*0201/*0202) and HLA-DQ8 (A1*0301, B1*0302) was used (GenID, GMBH, Strasburg, Germany). HLA-DQ2.5 haplotype was present in 24% of healthy

controls and 90% of CD patients in our geographical area.[2] In the present study, positive coeliac genetics indicated the presence of HLA-DQ2.5 and/or HLA-DQ8.

References:

1. Fernández-Bañares F, Carrasco A, García-Puig R, et al. Intestinal intraepithelial lymphocyte cytometric pattern is more accurate than subepithelial deposits of anti-tissue transglutaminase IgA for the diagnosis of coeliac disease in lymphocytic enteritis. PLoS One 2014;9:e101249.
2. Farré C. Enfermedad celíaca: Marcadores serológicos y de predisposición genética, aspectos clínicos y poblaciones de riesgo. Doctoral Thesis. University of Barcelona; 2002.

Table S1. Initial clinical picture in patients with or without response to GFD (*n* = 116).

	**Response GFD** *n* **= 62**	**Non-Response GFD** *n* **= 54**
IBS	11	9
Chronic diarrhoea	17	16
Chronic diarrhoea + Dyspepsia	6	2
Chronic diarrhoea + IDA *	3	2
Chronic diarrhoea + failure to thrive	3	1
Chronic diarrhoea + autoimmune disease	1	1
Dyspepsia + bloating	9	12
Dyspepsia + bloating + IDA	1	4
Dyspepsia + bloating + autoimmune disease	1	4
Dyspepsia + bloating + osteopenia	2	0
IDA	2	2
Failure to thrive	3	0
Hypertransaminasaemia	3	0
Other	0	1

* IDA, iron-deficiency anaemia

Table S2. Accuracy of the different criterion values and coordinates of the ROC curve.

**In all included patients (*n* = 104):**						
**Criterion**	**Sensitivity**	**95% CI**	**Specificity**	**95% CI**	**+LR**	**-LR**
≥0	100	92.9–100	0	0–6.6	1	
>0	100	92.9–100	3.70	0.5–12.7	1.04	0
>3	100	92.9–100	7.41	2.1–17.9	1.08	0
>4	100	92.9–100	16.67	7.9–29.3	1.20	0
>5	96	86.3–99.5	42.59	29.2–56.8	1.67	0.094
>6	96	86.3–99.5	44.44	30.9–58.6	1,73	0.090
>7	94	83.5–98.7	53.70	39.6–67.4	2.03	0.11
>8	88	75.7–95.5	81.48	68.6–90.7	4.75	0.15
>10	86	73.3–94.2	85.19	72.9–93.4	5.81	0.16
>12	70	55.4–82.1	92.59	82.1–97.9	9.45	0.32
>15	40	26.4–54.8	100	93.4–100		0.60
>16	36	22.9–50.8	100	93.4–100		0.64
>17	32	19.5–46.7	100	93.4–100		0.68
>18	30	17.9–44.6	100	93.4–100		0.70

>21	6	1.3–16.5	100	93.4–100		0.94
>25	0	0–7.1	100	93.4–100		1.00

**In seronegative patients (*n* = 84):**						
**Criterion**	**Sensitivity**	**95% CI**	**Specificity**	**95% CI**	**+LR**	**-LR**
≥0	100	88.4–100	0	0–6.6	1	
>0	100	88.4–100	3.70	0.5–12.7	1.04	0
>3	100	88.4–100	7.41	2.1–17.9	1.08	0
>4	100	88.4–100	16.67	7.9–29.3	1.20	0
>5	93.3	77.9–99.2	42.59	29.2–56.8	1.63	0.16
>6	93.3	77.9–99.2	44.44	30.9–58.6	1.68	0.15
>7	90	73.5–97.9	53.70	39.6–67.4	1.94	0.19
>8	80	61.4–92.3	81.48	68.6–90.7	4.32	0.25
>10	76.6	57.7–90.1	85.19	72.9–93.4	5.18	0.27
>12	50	31.3–68.7	92.59	82.1–97.9	6.75	0.54
>15	0	0–11.6	100	93.4–100		1

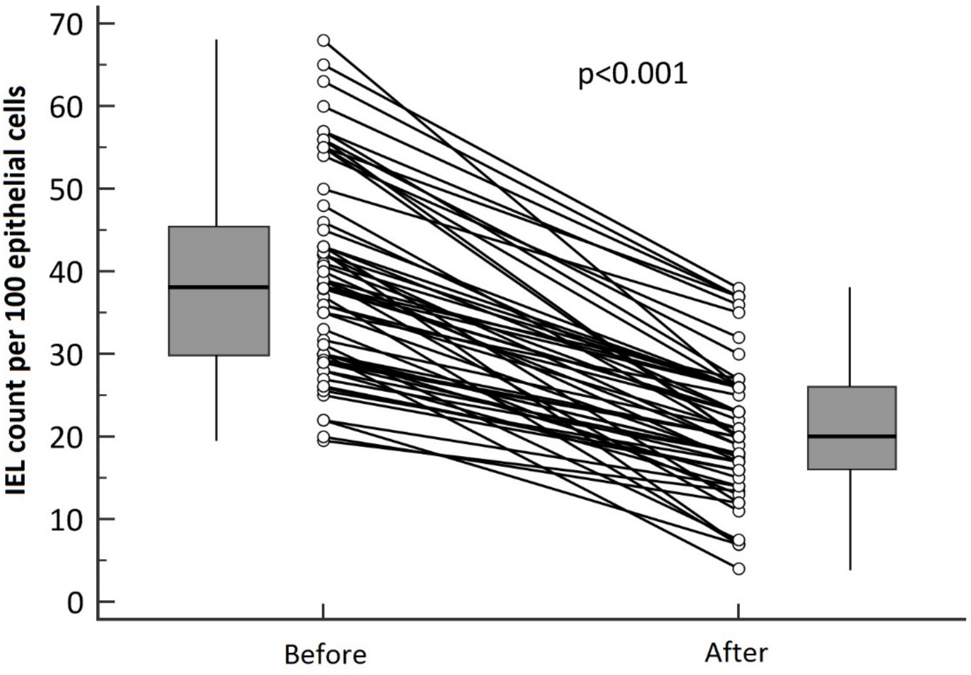

Figure S1. Evolution of the intraepithelial lymphocyte count (IEL count per 100 epithelial nuclei) in patients with clinical and histological remission/response after a GFD.

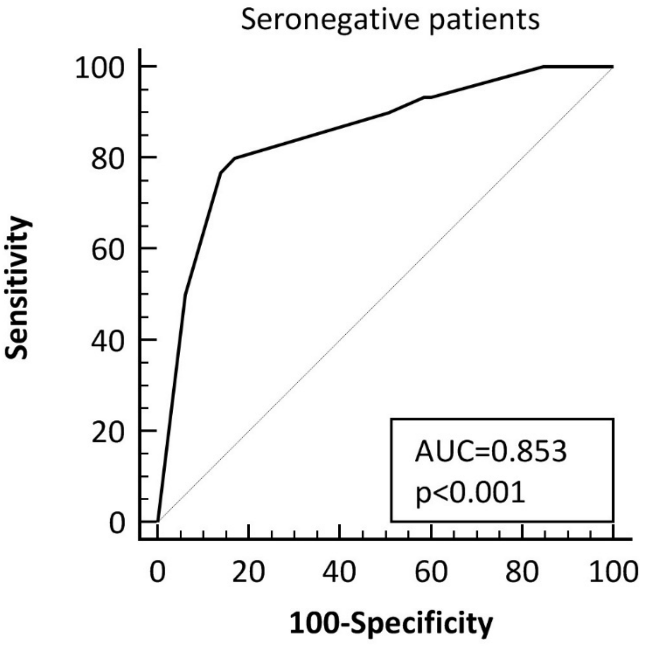

Figure S2. ROC curve for the accuracy of the scoring system at identifying patients with low-grade coeliac enteropathy after excluding patients with positive serum anti-tTG2. AUC: area under the curve, ROC: receiver operating characteristic.
